# Une tumeur de la valve mitrale

**DOI:** 10.11604/pamj.2018.30.15.13649

**Published:** 2018-05-08

**Authors:** Chahdi Hafsa, Damiri Amal, Oukabli Mohamed, Abderrahman Al Bouzidi

**Affiliations:** 1 Service d’Anatomie et Cytologie Pathologique, Hôpital Militaire d’Instruction Mohammed V, Rabat, Maroc

**Keywords:** Fibroélastome papillaire, tumeur, Valve mitrale, Papillary fibroelastoma, tumor, mitral valve

## Abstract

Le fibroélastome papillaire est une tumeur cardiaque primitive bénigne rare pouvant entraîner des complications emboliques sévères. Nous rapportons un cas chez un homme de 69 ans. C’est dans le cadre du bilan étiologique d’une dyspnée que l’échocardiographie transthoracique a montré une masse sessile sur la petite valve mitrale. Cette masse a été excisée chirurgicalement et analysée histologiquement. Le diagnostic de fibroélastome papillaire était alors confirmé.

## Abstract (en)

Papillary fibroelastoma is a rare primary benign cardiac tumor which can cause severe embolic complications. We report the case of a 69-year old man presenting with dyspnea. The patient underwent transthoracic echocardiography that showed sessile mass on the mitral valve. Surgically excision of the mass was performed followed by histological examination. The diagnosis of papillary fibroelastoma was then confirmed.

## Introduction

Le fibroélastome papillaire est une tumeur cardiaque primitive bénigne. Il représente environ 7 % des tumeurs cardiaques primitives [1]. Avant l’apparition de l’échographie, le diagnostic était occasionnel lors d’une chirurgie cardiaque ou d’une autopsie [1,2]. Le fibroélastome prend origine à partir de l’endocarde [1-4]; il est habituellement asymptomatique mais il peut également être à l’origine des complications graves telles que les accidents ischémiques cérébraux, les syndromes coronariens aigus, l’embolie pulmonaire et la mort subite. Nous rapportons un cas de fibroélastome papillaire de la petite valve mitrale découvert chez un homme âgé de 69 ans, sans facteur de risque cardiovasculaire, qui a présenté une dyspnée d’évolution progressive.

## Patient et observation

Il s’agissait d’un patient âgé de 69 ans, sans antécédents pathologique notables, qui a présenté une dyspnée d’aggravation progressive associée à une sensation de brouillard devant les yeux et une lipothymie. L’examen clinique trouvait un patient en bon état général, les bruits du cœur étaient réguliers, avec un souffle systolique au foyer aortique irradiant vers les vaisseaux du cou, l’examen pleuro-pulmonaire et le reste de l’examen somatique étaient sans particularités. La radiographie du thorax montrait une silhouette cardiaque de taille normale et une bonne transparence parenchymateuse L’électrocardiogramme était sans particularités.

**Figure 1:**
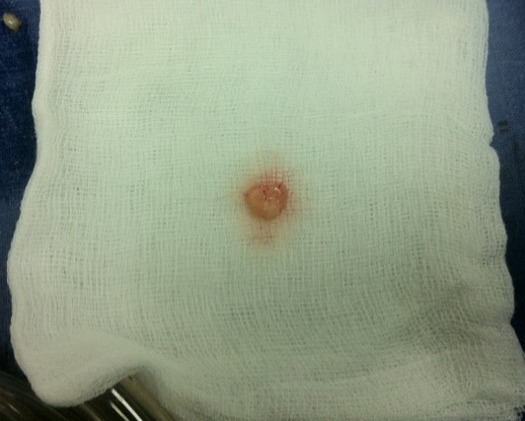
Aspect macroscopique de la tumeur réséquée

**Figure 2:**
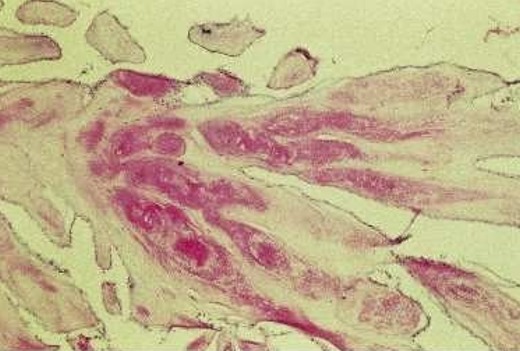
Formations papillaires tapissées par un revêtement endothélial régulier reposant sur un axe conjonctif hyalinisé au centre (coloration hématéine-éosine)

L’échocardiographie transthoracique a objectivé un rétrécissement aortique calcifié serré, un ventricule gauche de taille normale et de fonction systolique conservée. Au niveau de la valve mitrale on notait la présence d’une masse de 15mm de diamètre, s’insérant sur la petite valve mitrale sessile, sans effet de sténose ni de fuite mitrale. Le patient a bénéficié d’une coronarographie préopératoire qui était normale et le bilan biologique était sans particularités. Le patient a été opéré avec une stérnotomie médiane verticale, objectivant une masse de 15mm de diamètre sur la face auriculaire de la petite valve mitrale, friable et facilement clivable (Figure 1). La pièce opératoire a été adressée au laboratoire pour examen anatomo-pathologique. A l’étude microscopique il s’agissait d’une lésion tumorale d’architecture papillaire bordée par un revêtement endothélial régulier reposant sur un axe conjonctif hyalinisé et focalement œdémateux (Figure 2). Le diagnostic retenu est celui de fibroelastome papillaire de la valve mitrale.

## Discussion

Le fibroélastome papillaire est une tumeur cardiaque primitive bénigne. Il représente environ 7% des tumeurs cardiaques primitives et il est le troisième par ordre de fréquence après le myxome et le lipome [1,2]. Il prend naissance le plus fréquemment à partir de l’endocarde valvulaire. La surface valvulaire est la localisation prédominante (77% des cas). La valve aortique est la plus atteinte (30% des cas) suivie par la valve mitrale (20-25% des cas) [1]. Bien que le fibroélastome touche toutes les tranches d’âge depuis la période néonatale jusqu’à la dixième décennie de la vie, il prédomine chez l’adulte. L’âge moyen est de 60 ans. Le sex-ratio est supérieur à 1 [2,3]. La plupart des cas sont acquis mais d’étiologie inconnue [2,3]. Tous les cas décrits sont sporadiques, il n’y avait aucune forme familiale [1].

Macroscopiquement, il se présente comme une anémone de mer. Sa base d’implantation est pédiculée. Son corps forme de nombreux replis. Sa taille peut varier de deux millimètres à sept centimètres [2-4]. Sur le plan histologique, il est tapissé d’une monocouche de cellules endothéliales. Le tissu conjonctif sous-jacent est riche en fibres collagènes, fibres élastiques, glycosaminoglicanes et cellules musculaires lisses [4]. Les diagnostics différentiels du fibroélastome papillaires se posent surtout sur le plan clinique avec le thrombus intracardiaque, les autres tumeurs, bénignes ou plus rarement malignes et les végétations dans le cadre d’une endocardite infectieuse. Le contexte clinique, l’évolution sous traitement anticoagulant et/ou antibiotique et les données d’imagerie sont nécessaires pour évoquer le diagnostic [2,5]. L’exérèse chirurgicale est le traitement de référence, le plus souvent en conservant la valve native [2-5]. Pour notre patient les suites opératoires immédiates étaient simples avec un état hémodynamique stable et un séjour à la réanimation ne dépassant pas 48 heures.

## Conclusion

Malgré que le fibroélastome soit une tumeur histologiquement bénigne, son évolution peut être marquée par des complications fatales comme les embolies systémiques et pulmonaires, et la mort subite. Son traitement est chirurgical par une excision de la tumeur. Le pronostic postopératoire est excellent et le risque de récidive tumorale est faible.

## Conflits d’intérêts

Les auteurs ne déclarent aucun conflit d’intérêts.
